# Effect of Antimicrobial Peptide, Nisin, on the Reproductive Functions of Rats

**DOI:** 10.5402/2011/828736

**Published:** 2012-01-11

**Authors:** K. V. R Reddy, S. M. Gupta, C. C. Aranha

**Affiliations:** Department of Molecular Immunology, National Institute for Research in Reproductive Health, J. M. Street, Parel, Mumba 400 012, India

## Abstract

Our previous studies have demonstrated that naturally occurring peptide, Nisin possess antibacterial activity and did not interfere with rabbit vaginal mucosa. In this study, the reproductive toxicity of the Nisin in male rats was evaluated. Rats were fed orally with Nisin (10, 25, and 50 mg/kg/day) for 13 weeks. No treatment related mortality was observed. The body weight gain, food consumption and serum biochemical parameters were at par with the control group. Histomorphology of the selected reproductive (testis, epididymis, ventral prostate, and seminal vesicle) and nonreproductive (liver and kidney) tissues was observed to be normal. There was no treatment-related increase or decrease in the expression of testis-specific genes (c-Kit, GATA-1, and HILS-1) and the activity levels of epididymal *α*-glucosidase, ventral prostate alkaline phosphatase (AlP), liver alanine aminotransferase (AlAT) and aspartate aminotransferase (AAT). Fructose and lactic acid levels in the seminal vesicles also remained unchanged. These studies suggest that Nisin did not affect the normal physiology of these organs. In addition, no adverse effects were observed on the reproductive performance of Nisin-treated male rats and their offspring. In conclusion, the current studies support our earlier studies, which demonstrated suitability of Nisin as a safe and effective microbicide.

## 1. Introduction

Microbicides are now recognized as the most promising prevention technologies to combat the current pandemic of sexually transmitted infections (STIs) including AIDS [1, 2]. Several anionic polymers and detergent-based microbicides are undergoing preclinical development, but their long-term mucosal safety is a cause for concern [3, 4]. Frequent use of Nonoxynol-9 (N-9) is known to cause inflammation and lesions in the cervicovaginal epithelium and alteration in vaginal microbial milieu, leading to increased risk of contracting STIs [5]. Therefore, the immediate priority lies in the development of safe, efficacious, non-detergent-type mechanism-based microbicides.

A group of compounds exhibiting the above properties are naturally occurring antimicrobial peptides (AMPs). One of the well-characterized AMPs is Nisin, a 34 amino acid cationic, amphiphilic peptide belonging to the lantibiotic family [6]. The peptide is produced by certain strains of *Lactococcus lactis*. Nisin has been used as a food preservative for the last five decades [7, 8] and is being evaluated for its efficacy as a vaginal microbicide. Since any microbicide developed for intravaginal use is likely to be used for anal sex by men and women, it is essential to ensure its safety. Our earlier studies have shown that even high doses of Nisin does not induce any abnormalities in the vaginal cell morphology nor does it affect the structural integrity of vaginal epithelium in rabbits [9].

The present study was designed to evaluate (1) the cumulative toxicity of Nisin in male rats, determine its metabolic tolerance, and also ascertain its risk/benefit profile and (2) determine the effect of Nisin on general health and reproductive performance in male rats. 

## 2. Materials and Methods

### 2.1. Materials

Nisin (Sigma St. Louis, USA) was purified as described earlier [10] and used in the present study.

### 2.2. Animals

Mature, 70-day-old male (mean body weight 140 ± 10 g) Holtzman strain rats (mean body weight, 120 ± 10 g) were used in the present study. The animals were maintained in our animal facility at a constant 12 : 12 dark : light cycle and controlled temperature (22 ± 2°C) with a relative humidity of 50%. Standard pellet food and tap water were available *ad libitum*. The study was approved by the Animal Ethics Committee of the National Institute for Research in Reproductive Health (NIRRH), Parel, Mumbai (IAEC no. 1/99) and the Committee for the Purpose of Control and Supervision of Experiments on Animals (CPCSEA), Ministry of Social Justice and Empowerment, Government of India.

### 2.3. Experimental Procedures

#### 2.3.1. Oral Administration of Nisin in Male Rats

Adult male rats were randomly divided into six groups: three controls and three treated, consisting of 10 animals per group. Nisin was administered by oral gavage, once daily at 10, 25, and 50 mg doses per kg body weight for 13 consecutive weeks. Since the parameters studied did not differ among the three groups, the results obtained with 50 mg dose are discussed in this study.

#### 2.3.2. Body and Organ Weights

Body weights, clinical and behavioral changes of control and treated rats (10, 25 and 50 mgs/kg/day) were recorded daily during the treatment period. Control animals received only the vehicle (0.9% saline). On each data point the mean body weights of all the three treatment groups was plotted. On day 91, five animals from each group were sacrificed. Prior to autopsy, 3-4 mL of blood was collected into heparinized tubes by cardiac puncture for hematological and serum biochemical analysis. The vital nonreproductive (liver and kidney) and reproductive (testis, epididymis, ventral prostate, and seminal vesicles) tissues were excised, cleared of fat, connective tissues, and weighed to the nearest milligram. All organs were stored at −80°C until processed for histological and biochemical analysis.

The remaining five rats from each group were cohabited with proven fertile female rats in the proestrus-estrus transition phase. The mated females rats (*n* = 10) were allowed till term. The duration of gestation, number of pups, and weight of pups born were recorded. General condition of the offspring was monitored for a period of 3 weeks to determine the perinatal effect of Nisin.

#### 2.3.3. Histological Evaluation of Reproductive and Nonreproductive Tissues of Rat

The tissues such as testis, epididymis, ventral prostate, seminal vesicle, liver, and kidney were isolated from five rats each of the control and treated groups. A portion of each tissue was fixed in Bouin's solution. Paraffin sections of 5 *μ*m thick were cut and stained with hemtoxylin and eosin (Ha and Ea). The sections were evaluated semiquantitatively to account for incidental histopathological lesions.

#### 2.3.4. Sperm Production, Sperm Transit, and Sperm Morphology

After removal of tunica albuginea, the testis was minced and homogenized in 10 mL of NaCl containing 0.5% Triton-X100 and centrifuged at 600 ×g for 1 min. The number of homogenization-resistant spermatids was counted in a hemocytometer. Daily sperm production (DSP) was derived by dividing the total number of homogenization-resistant spermatids per testis by 6.1 days (the number of days of a seminiferous cycle in which the spermatids are present). Also, transit time in days through each of these regions was calculated by dividing the epididymal sperm number by DSP. To assess the percentage of morphologically abnormal sperm, the vas deferens was rinsed with 0.5 mL of physiological saline (0.9% NaCl) to obtain a sperm suspension. Aliquots of sperm suspension were stained with 2% eosin. Two hundred spermatozoa per animal were analyzed microscopically at 400x magnification and spermatozoa with abnormal heads and/or abnormal tails were scored.

#### 2.3.5. Epididymal Sperm Count

Epididymal sperm count of the control and treated animals was determined by the method as described in the WHO manual [11, 12]. Briefly, to isolate epididymal sperm, caput, corpus, and cauda epididymis were cut into small pieces and suspended in Ham's F-10 medium. An aliquot of epididymal sperm (5 *μ*L) was diluted with 95 *μ*L of diluent (2.5 g sodium bicarbonate, 500 *μ*L of 35% formalin, and 12.5 mg of trypan blue in 50 mL of distilled water) and counted using Neubauer hemocytometer.

#### 2.3.6. Hematological Parameters

The hematological parameters were analyzed using a multiparameter, automated analyzer (Swelab instruments, Stockholm, Sweden), which was standardized for rat blood [10] and used for the measurement of RBCs, total leucocytes (neutrophils, basophils, lymphocytes, monocytes, and eosinophils), and hemoglobin concentration.

#### 2.3.7. Clinical Chemistry Profiles

Biochemical analysis of blood plasma samples was performed spectrophotometrically (Shimadzu-UV 160, Japan). The levels of total proteins, albumin, creatinine, uric acid, blood urea nitrogen, total cholesterol, glucose, phosphorus, sodium, potassium, chloride, calcium, alanine aminotransferase (AlAT) (EC: 2.6.11), and aspartate aminotransferase (AAT) (EC: 2.6.1.2) [13] were determined.

#### 2.3.8. Enzyme Activities

Extracts of each of the accessory reproductive organs were prepared by homogenizing preweighed tissues in distilled water. Protein levels were determined by Folin-Ciocalteu method [14]. The enzyme assays were conducted under the conditioned following zero-order kinetics after preliminary standardization regarding linearity with respect to time of incubation and enzyme concentration. The activity levels of epididymal *α*-glucosidase (EC: 3.2.1.20) [15], ventral prostate alkaline phosphatase (EC: 3.1.3.1) (AlP) [16], and lactic acid [17] and fructose levels [18] from seminal vesicles were measured spectrophotometrically using standard methods given in the parentheses.

#### 2.3.9. RNA Isolation and RT-PCR

After mating studies, the control and treated male rats were autopsied, testes collected aseptically and RNA was isolated using Qiagen RNA extraction kit and Trizol reagent [19]. Briefly, RNA was quantitated spectrophotometrically, at 260 and 280 nm. The total RNA (1 *μ*g) was reverse-transcribed into single-stranded cDNA using superscript II reverse transcriptase. Control cDNA was prepared without reverse transcriptase. Amplification of testicular DNA was carried out by RT-PCR using gene-specific primers for c-Kit protooncogene (Gene ID-NM_021099; 5132 bp, flanking sequence: 450–471 and 852–831 bp on mRNA); (GATA-1) (Gene ID-NM_008089; 1807 bp mRNA, flanking sequence: 678–700 and 919–903); histone-like linker protein (HILS1) (Gene ID NM_018792) (796 bp mRNA, flanking sequence 133–154 and 350–329) and housekeeping gene, cyclophilin-A (CYP-A) (Gene ID-NM_008907 (736 bp mRNA, flanking sequence 124–147 and 588–565 bp on mRNA) (Table 1).

The total reaction mixtures (50 *μ*L) were heated at 96°C for 1 min and immediately carried through 2 cycles of PCR consisting of a denaturing step of 1 min at 96°C and an extension step of 4 min at 58°C and then through 30 cycles, each with denaturing step of 1 min at 94°C and extension step of 2.5 min at 58°C. The final extension was performed at 70°C for 10 min. As negative control, reactions were carried out using samples without RNA and with cDNA prepared in the absence of reverse transcriptase enzyme. The amplified products were separated on a 2% agarose gel, which was subsequently stained with ethidium bromide (Sigma, St. Louis, USA) and photographed under UV illumination using the Bio-Rad video capture gel documentation system-1000 (Bio-Rad, Richmond, CA, USA). Densitometric scanning was performed with a Bio-Rad model GS-700 imaging densitometer and the data was analyzed with Image 1.62 software and normalized to those of CYP-A.

#### 2.3.10. Reproductive Performance of Male Rats Exposed to Nisin

To determine the fertility and reproductive performance, Nisin-treated male rats were cohabited with proven fertile females. The mated female rats were monitored for duration of gestation and weight gain in the proestrus-oestrus transition phase during the first week of the treatment. At the end of gestation the litter size, number of dead fetuses, weight of pups, and their growth were recorded.

#### 2.3.11. Reproductive Capacity of F1 Male Mice

To examine the reproductive ability of male mice born to Nisin-treated parental males, F1 males were mated with normal healthy female mice. Total number of pups born per mother and sex ratio were recorded.

#### 2.3.12. Statistical Analysis

Repeated measures of body weight gain were determined by the analysis of covariance (ANCOVA) between the data points of the control and treated groups. Food consumption, hematological and serum biochemical profiles were evaluated by one-way Analysis of Variance (ANOVA) [20]. Differences in the sperm count, sperm transit rate and various biochemical parameters were analyzed by student's *t*-test. Means were considered significantly different when *P* < 0.05.

## 3. Results

### 3.1. Effect of Oral Administration of Nisin for 13 Weeks on General Health and Reproductive Performance of Rats

**Figure 1:**
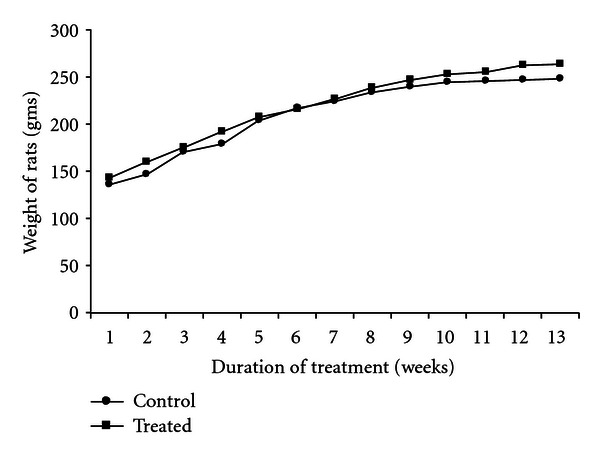
Body weight changes in control and Nisin-treated rats. Each data point is the mean of all the three treatment groups per week. No significant difference was observed in the body weights between control and Nisin-treated groups as determined by analysis of covariance (ANCOVA) between the data points of the control and treated groups.

The toxicological profile of Nisin was evaluated by monitoring its effects on general health and behavior, weight changes, hematology, serum biochemical parameters, functional efficiency of different tissues, and reproductive performance. Repeated oral administration of Nisin (10, 25 and 50 mg/kg) for 13 consecutive weeks revealed no adverse effects in treated rats. Mortality did not occur, and all the animals were clinically healthy at the end of the study. Mean body weight gain and final mean body weight of the rats did not differ during the treatment period and were similar to the controls (Figure 1). No statistically significant treatment-related differences were seen in the absolute weights of testis, epididymis, ventral prostate, and seminal vesicle in Nisin-dosed rats (Table 2).

**Figure 2:**
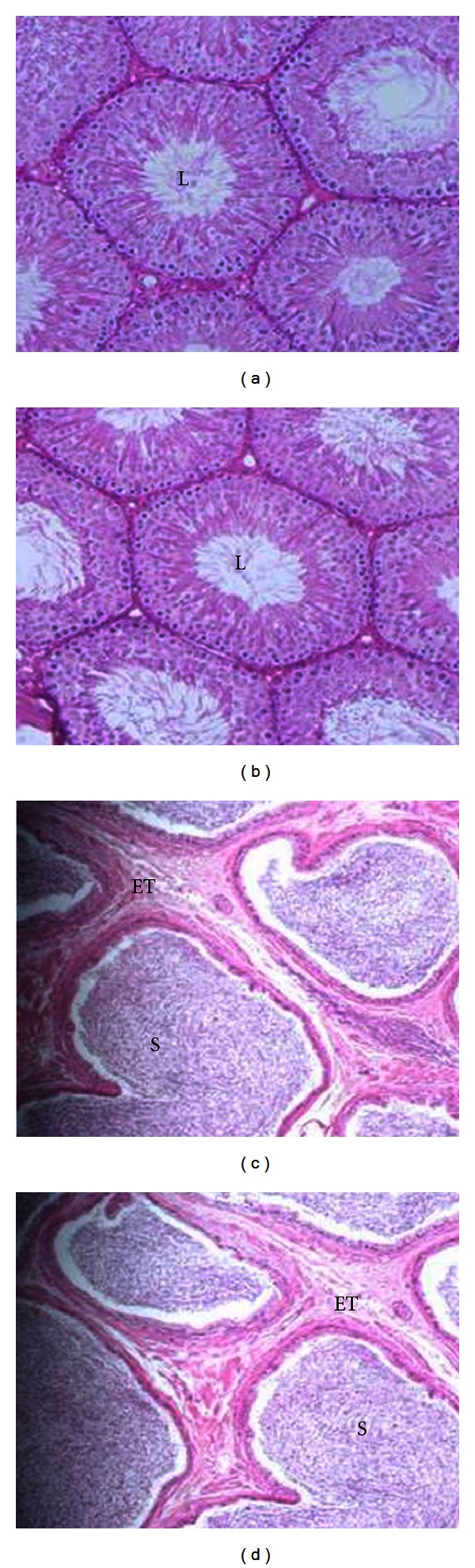
Effect of oral administration of Nisin (50 mg/kg/day) for 90 days on structural organization of testis (a, b) and epididymis (c, d) in control (a, c) and treated (b, d) rats. No treatment related abnormalities were observed. S: Epididymal sperm, L: Testicular lumen, and ET: Epididymal tubule.

Histological evaluation of testis, epididymis, ventral prostate, and seminal vesicle revealed no gross changes in the morphology of testicular parenchyma, diameter of the seminiferous tubules/lumen, and thickness of the epididymal epithelium (Figure 2). The sperm number in the cauda epididymis, daily sperm production, sperm morphology, and sperm transit rate was not altered (Table 3). Incidental histological lesions which included mild inflammation of the kidney and liver (normally expected to be seen in the laboratory rat) were distributed randomly in control and treated groups. No focal interstitial inflammatory lesions and tubular atrophy were noted. In general these studies suggest that Nisin does not cause any histopathological abnormalities in the reproductive and nonreproductive tissues.

#### 3.1.1. Hematological and Serum Biochemical Parameters

The values of hematological parameters (total RBC count, haemoglobin concentration, and haematocrit) and white blood cells (lymphocytes, neutrophils, eosinophils, and basophils) were within the normal limits, and no biologically significant differences were observed between the control and treated groups. Analysis of serum biochemical parameters also did not reveal any treatment-related changes (Table 4). The kidney function (BUN and CRE), liver function (AlAT, AAT, cholesterol, and triglycerides), pancreas function (amylase and glutamine), immunological function (globulin), nutritional status (total proteins, calcium, phosphorous), and plasma electrolytes (sodium and potassium) were also not altered by the treatment (Table 5).

#### 3.1.2. Substrate and Enzyme Activity Levels

The concentrations of lactic acid, fructose and the activity levels of *α*-glucosidase of epididymis and alkaline phosphatase of ventral prostate did not reveal changes in any of these parameters when compared to their respective controls (Table 6).

#### 3.1.3. Expression of Testis-Specific Genes in Rats

**Figure 3:**
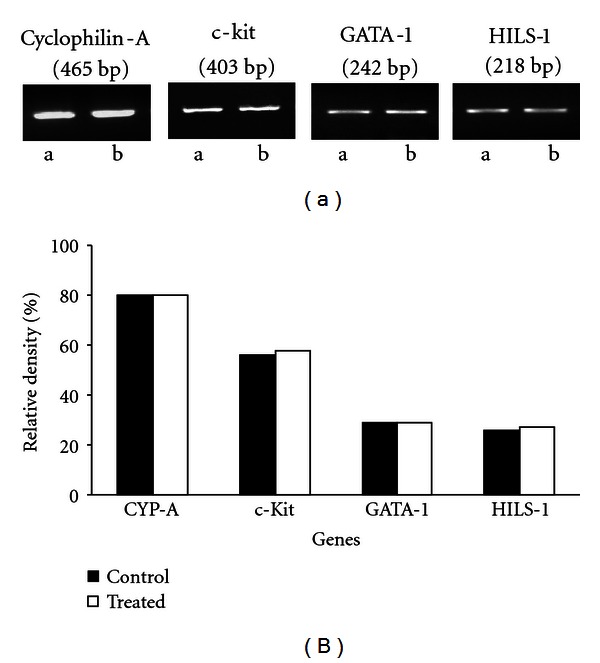
(a) RT-PCR analysis of testicular germ-cell-specific marker genes in the control (a) and Nisin-treated (b) animals as described in Section 2. (b) A quantitative assessment of the intensity of each band was determined by densitometry. No significant difference was observed in the expression of genes between the groups.

Germ-cell-specific expression of different genes has been studied in the testis of control and Nisin-treated rats. The expression pattern of testicular germ-cell marker genes such as c-Kit (spermatogonial cells), GATA-1 (Sertoli cells), and HILSI (spermatids) was not altered in Nisin-treated animals and was found to be similar to the controls (Figure 3).

#### 3.1.4. Reproductive Performance of Male Rats Exposed to Nisin

To assess the reproductive performance, rats treated with the highest dose of Nisin (50 mg/kg) were allowed to mate with proven fertile females. The mated female showed study increase in weight, and gestation period was also normal. No significant differences in litter size, weight, and general health of pups was noted with no perinatal or postnatal repercussions (Table 7). Growth of the pups was observed until the end of lactation and found to be similar to the control group.

#### 3.1.5. Fertility Status of F1 Progeny

Nisin treatment did not induce any adverse effects on the subsequent fertility of the F1 progeny of both male and female offspring, born to the treated parental males when compared to the offspring born to the control animals. Total number of pups born per mother and sex ratio were not different between the two groups (data not shown).

## 4. Discussion

In the present study, we have made an attempt to demonstrate the effect of Nisin on general health and reproductive performance of male rats. Nisin, a 34 amino acid naturally occurring peptide, has been shown to demonstrate antimicrobial and spermicidal properties [10]. Due to these activities, it becomes a suitable alternative for detergent-based microbicides that are known to have adverse effects. For the development of any compound as a microbicide, it requires that the compound be tested for toxicity in suitable animal models to validate its safety [21, 22]. Hence we evaluated the cumulative toxicity of Nisin in order to determine its metabolic tolerance and ascertain its effect on reproductive function in rats. Repeated oral administration of 10, 25, and 50 mg of Nisin in rats (50, 125, and 250 times higher, resp. than its *in vivo* contraceptive dose) for 13 weeks did not cause treatment-related mortality or morbidity. Though, Nisin is being used as a natural and safe food preservative, it is essential to determine its safety while it is being developed for an alternative use, that is, vaginal microbicide [21].

Earlier reports by Hara and colleagues [23] evaluating the safety of Nisin as a food preservative in male rats treated for 13 consecutive weeks indicated that rats fed orally with commercial Nisin (0.0125–125 *μ*g/kg/day) did not affect the growth and histomorphology of various tissues. In another study, similar observations were made when higher doses of Nisin (15–20 mg/kg/day) were administered [24]. In these studies commercial Nisin supplied with milk solids and high salt concentration (77%) was used. However in the present study, RP-HPLC purified Nisin free from the above ingredients was used at a higher concentration than doses tested previously.

Subchronic exposure of rats to Nisin (10, 25, 50 mg/kg/day) for 13 weeks did not cause any increase and/or decrease in body and organ weights. Physical parameters such as weights of epididymis, ventral prostate and seminal vesicle, liver and kidney have been considered as gravimetric markers to assess metabolic efficiency of these organs [25], which depends on internal and external environmental factors such as feed, room temperature, and humidity. No changes in absolute and relative weights of these organs were observed at the end of treatment period. Since changes in organ weights are apparent only after substantial damage to the tissues, reliance on more sensitive endpoints such as morphological and functional abnormalities of these tissues could be helpful to better delineate the alterations. The hematological and serum biochemical profiles also did not deviate from the control values, suggesting normal function of various systems in the body.

Alkaline phosphatase (ALP) was analyzed as it plays an important role in the synthesis of fructose, an essential constituent of seminal plasma in ventral prostate [26, 27]. The ALP levels did not differ between the control and treated groups, suggesting normal functioning of the prostate. The activity levels of *α*-glucosidase, a marker for epididymal sperm maturation [28], and lactic acid another important seminal plasma constituent of seminal vesicle [27] were also analyzed and remained unchanged, suggesting normal synthesis of seminal plasma constituents by the accessory reproductive organs after Nisin treatment.

Alanine aminotransferase (AlAT) and aspartate amino transferase (AAT) play an important role in the mobilization of amino acids into gluconeogenesis in the liver and kidney [29]. The activity levels of both these enzymes did not show any alteration after Nisin treatment, indicating normal functional efficiency of liver and kidney.

Since Nisin is known to be absorbed systemically and accumulate in vital organs in the body [9], its effect on the functions of different tissues was evaluated. The results indicated no treatment-related alterations in the structural integrity of the reproductive (testis, epididymis, ventral prostate, seminal vesicle) and nonreproductive (liver and kidney) after Nisin treatment compared to their respective controls (Figure 2).

In order to determine the effect of Nisin on spermatogenesis at the molecular level, we studied the expression of a battery of cell- and stage-specific genes in the testes. Of the genes selected, c-Kit, GATA-1, HILS-1 genes play a key role in the differentiation of testicular germ cells. c-Kit a spermatogonial-cell-(SGC-) specific marker is required for germ-cell migration, proliferation, and survival [30]. GATA-1 is a transcription factor, specifically expressed by the Sertoli cells [31]. Linker histone H1-like spermatid-specific gene (HILS1) has been implicated in chromatin remodeling and DNA repair which is critical for the formation of functional sperm [32]. The results indicated that there was no change in the expression of these genes after Nisin treatment, suggesting normal differentiation of germ cells into mature spermatozoa (Figure 3). The results showed normal sperm production and sperm transit rate in Nisin-treated rats. The number of spermatozoa in the cauda epididymis also remained unchanged and the values were at par with the controls, suggesting noninterference of Nisin in the spermatogenic process. Moreover, in F1 generation, the total number of pups per litter and the sex ratio were not different when the Nisin-treated parental males were mated with normal females.

## 5. Conclusions

The present data along with our previous studies suggest that Nisin does not affect the general health and reproductive performance of male rats. These observations are strongly supported by normal function of reproductive as well as nonreproductive tissues and unaltered expression of genes involved in spermatogenesis.

## Figures and Tables

**Table 1:** Sequences of oligonucleotide primers used for PCR.

Gene	Product size (bp)	Primer sequences	Annealing temperatures
C-Kit	403	F: 5′-ACC CAC AGG TGT CCA ATT ATT C-3′ R: 5′-TGG CGT TCA TAA TTG AAG TCA C-3′	60°C

GATA-1	242	F: 5′-TGT GTG AAC TGT GGA GCA ACG GC-3′ R: 5′-AAA TGA AGG CCG CAG GCA TTG CA-3′	62°C

HILS1	218	F: 5′-TGG AGT ATC TAG CAC CTG GAG T-3′ R: 5′-TCA GAA TCA CAT ACG ACA TGG T-3′	62°C

Cyclophilin-A	465	F: 5′-AAG TTC CAA AGA CAG CAG AAA ACT-3′ R: 5′-GAG CTA CAG AAG GAA TGG TTT GAT-3′	59°C

**Table 2:** Weight changes (mg) in various reproductive organs of male rats exposed orally with Nisin (50 mg/kg/day) for 90 days. Each value is the mean ± S.D. of five observations. No significant difference (*P* > 0.05) was recorded between the groups.

	Testis	Epididymis	Ventral prostate	Seminal vesicle
Control	1710 ± 263	901 ± 134	320 ± 36	738 ± 65
Treated	1805 ± 318	879 ± 92	333 ± 29	711 ± 54

**Table 3:** Effect of oral exposure of male rats with Nisin (50 mg/kg/day) for 90 days on daily sperm production, sperm number in cauda epididymis, sperm transit rate and sperm morphology was determined as described in Section 2. Each value is the mean ± S.D. of five observations. No significant difference was recorded between the groups.

	Daily sperm production	Sperm number	Sperm transit rate	Abnormal sperm (%)
Control	40.32 ± 3.42	286 ± 37	6.34 ± 1.97	1.22 ± 0.13
Treated	40.63 ± 4.14	280 ± 30	6.22 ± 2.13	0.99 ± 0.10

**Table 4:** Effect of oral exposure of Nisin (50 mg/kg/day) on hematological profiles in male rats. Each value is the mean ± S.D. of five observations. No significant difference was observed between the control and treated groups.

Parameter	Control	Treated
RBC (×10^6^/mm^3^)	4212 ± 192	4298 ± 236
Hematocrit (%)	36.88 ± 3.14	35.64 ± 2.11
Hemoglobin (g/dL)	11.99 ± 1.34	12.07 ± 0.91
WBC (×10^3^/mm^3^)	5008 ± 491	5063 ± 388
Neutrophils (%)	23.94 ± 1.00	24.01 ± 1.68
Monocytes (%)	2.63 ± 0.98	2.71 ± 0.34
Lmphocytes (%)	4.86 ± 0.27	4.77 ± 0.30
Eosinophils (%)	2.31 ± 0.37	2.68 ± 0.30

**Table 5:** Effect of oral exposure of Nisin (50 mg/kg/day) on serum biochemical profiles in male rats. Each value is the mean ± S.D. of five observations. No significant difference was observed between both the groups.

Parameter	Control	Treated
Total protein (g %)	5.17 ± 0.40	5.34 ± 0.63
Albumin (gm %)	1.21 ± 0.19	1.30 ± 0.28
Blood urea nitrogen (mg %)	12.17 ± 0.94	13.01 ± 1.86
Creatinine (mg %)	0.63 ± 0.10	0.71 ± 0.05
Glucose (mg %)	87.00 ± 4.14	90.32 ± 5.06
Uric acid (mg %)	2.11 ± 0.27	2.32 ± 0.30
Calcium (mg %)	7.87 ± 0.91	8.01 ± 1.07
Phosphorus (mg %)	3.07 ± 0.20	3.17 ± 0.19
Sodium (meq/L)	123.03 ± 12.11	121.18 ± 9.34
Potassium (meq/L)	3.11 ± 0.23	3.03 ± 0.18
Chloride (meq/L)	81.94 ± 7.03	83.15 ± 7.07
Alanine amino transferase (*μ*Mol of pyruvate formed/mg protein/hr)	108.04 ± 3.53	1.16.23 ± 1.94
Asparate amino transferase (*μ*Mol of pyruvate formed/mg protein/hr)	77.01.94 ± 9.03	76.32 ± 7.14
Total cholesterol (mg %)	99.04 ± 2.87	97.61 ± 2.58

**Table 6:** Effect of oral exposure of Nisin (50 mg/kg/day) on various biochemical parameters of reproductive tissues in male rats. Each value is the mean ± S.D. of six observations. No significant difference was observed between the two groups.

	Epididymis	Seminal vesicle	Ventral prostate		Liver		Kidney	
	*α*-glucosidase (1 nM of substrate hydrolyses/hr)	Lactic acid (mg/gm tissue)	Fructose (mg/gm tissue)	Alkaline phosphatase (*μ* mol Pi formed/mg protein/h)	AlAT	AAT	AlAT	AAT
					*μ*Mol of pyruvate formed/mg protein/hr		*μ*Mol of pyruvate formed/mg protein/hr	

Control	23.24 ± 1.18	2.17 ± 0.84	2.11 ± 0.76	30.34 ± 2.81	0.93 ± 0.07	0.67 ± 0.07	0.63 ± 0.05	0.51 ± 0.05
Treated	24.19 ± 1.16	2.04 ± 0.71	2.81 ± 0.88	30.18 ± 3.00	0.89 ± 0.08	0.69 ± 0.06	0.62 ± 0.05	0.46 ± 0.05

**Table 7:** Effect of oral exposure of Nisin (50 mg/kg/day) on the evaluation of fetal parameters. Number of male rats is in parentheses. Values are mean ± S.D. of ten pregnant rats. No significant differences were observed between the control and treated rats.

Male Rats	Pregnant females	Litter size	Dead fetuses	Weight of pups (g)
Control (5)	10	10.50 ± 0.20	0	2.98 ± 0.66
Treated (5)	10	11.0 ± 0.13	0	2.87 ± 0.51
